# Clinical and genetic analysis further delineates the phenotypic spectrum of *ALDH1A3*-related anophthalmia and microphthalmia

**DOI:** 10.1038/s41431-023-01342-8

**Published:** 2023-03-31

**Authors:** Yesim Kesim, Fabiola Ceroni, Alejandra Damián, Fiona Blanco-Kelly, Carmen Ayuso, Kathy Williamson, Véronique Paquis-Flucklinger, Dorine A Bax, Julie Plaisancié, Claudine Rieubland, Mostafa Chamlal, Marta Cortón, Nicolas Chassaing, Patrick Calvas, Nicola K Ragge

**Affiliations:** 1 Department of Biological and Medical Sciences, Faculty of Health and Life Sciences, Oxford Brookes University, Oxford, UK; 2 Department of Pharmacy and Biotechnology, University of Bologna, Bologna, Italy; 3 Department of Genetics & Genomics, Instituto de Investigación Sanitaria-Fundación Jiménez Díaz University Hospital, Universidad Autónoma de Madrid (IIS-FJD, UAM), Madrid, Spain; 4 Centre for Biomedical Network Research on Rare Diseases (CIBERER), Madrid, Spain; 5 MRC Human Genetics Unit, MRC Institute of Genetics and Molecular Medicine, University of Edinburgh, Western General Hospital, Edinburgh, UK; 6 Department of Medical Genetics, Nice Teaching Hospital, Nice, France; 7 INSERM U1214, ToNIC, Université Toulouse III, Toulouse, France; 8 Centre de Référence des Affections Rares en Génétique Ophtalmologique CARGO, Site Constitutif, CHU Toulouse, Toulouse, France; 9 Department of Human Genetics, Inselspital, Bern University Hospital, University of Bern, Bern, Switzerland; 10 Department of Pediatrics, RAZI-CLINIC Hospital, Tangier, Morocco; 11 Department of Clinical Genetics, West Midlands Regional Clinical Genetics Service and Birmingham Health Partners, Birmingham Women’s and Children’s Foundation Trust, Birmingham, UK

**Keywords:** Disease genetics, Neurodevelopmental disorders

## Abstract

Biallelic pathogenic variants in *ALDH1A3* are responsible for approximately 11% of recessively inherited cases of severe developmental eye anomalies. Some individuals can display variable neurodevelopmental features, but the relationship to the *ALDH1A3* variants remains unclear. Here, we describe seven unrelated families with biallelic pathogenic *ALDH1A3* variants: four compound heterozygous and three homozygous. All affected individuals had bilateral anophthalmia/microphthalmia (A/M), three with additional intellectual or developmental delay, one with autism and seizures and three with facial dysmorphic features. This study confirms that individuals with biallelic pathogenic *ALDH1A3* variants consistently manifest A/M, but additionally display neurodevelopmental features with significant intra- and interfamilial variability. Furthermore, we describe the first case with cataract and highlight the importance of screening *ALDH1A3* variants in nonconsanguineous families with A/M.

## Introduction

Anophthalmia (absence of visible ocular tissue) and microphthalmia (reduced ocular size) (A/M) are developmental eye anomalies affecting around 11.9 per 100,000 live births [1]. More than half of affected individuals exhibit variable extraocular features [2]. Pathogenic variants in at least 120 genes are known to underlie A/M, including several in the retinoic acid pathway: *STRA6, RBP4, RARB* and *ALDH1A3* [3]. ALDH1A3 (Aldehyde dehydrogenase 1 family member A3) catalyses retinoic acid formation, playing a key role in embryonic eye development [4]. Pathogenic biallelic variants in *ALDH1A3* are responsible for ∼11% of cases in consanguineous families [5–8]. To date, the majority of *ALDH1A3* variants are reported in individuals from consanguineous families and are consistently associated with bilateral A/M, with additional systemic features described in some cases [6, 8, 9]. However, genotype-phenotype correlations are unclear, with particular uncertainty surrounding whether the neurodevelopmental manifestations are solely linked to these variants.

Herein, we report nine cases with biallelic *ALDH1A3* variants from seven families. All affected individuals display bilateral A/M, with variable additional neurodevelopmental anomalies in some cases, providing further insights into the phenotypic spectrum.

## Materials and methods

We identified seven families from a cohort of 202 undiagnosed UK, French and Spanish families with A/M. Families 1, 2, 4 and 7 are from research studies: UK ‘Genetics of Eye and Brain anomalies’ (Cambridge East Ethics Committee (04/Q0104/12)), Deciphering Developmental Disorders (DDD) Study (Cambridge South Research Ethics Committee (10/H0305/83), Republic of Ireland (GEN/284/12)) and Genetics of Congenital Ocular Disorders, Fundación Jimenez Díaz University Hospital (Ethics Research Committee FJD (PIC015-18)), respectively. Families 3, 5 and 6 were identified through diagnostic testing. Informed consent was obtained from all individuals in accordance with the Declaration of Helsinki.

*ALDH1A3* (NM_000693.4) variants were identified from the cohort (*n* = 202) using whole genome (*n* = 20)/exome (*n* = 88) sequencing (WGS/WES), customized NGS panels (*n* = 91) and Sanger sequencing (*n* = 3). WGS/WES was performed using TruSeq Nano DNA Sample Prep (Illumina Inc., San Diego, CA, USA) and Agilent SureSelect Human All Exon V6 (Agilent Technologies, Santa Clara, CA, USA) kits, respectively. The majority of individuals (*n* = 181) received copy number variant screening using SNP-Array or array-CGH. WGS/WES data were annotated and filtered using an in-house pipeline. We used SIFT [10], Polyphen-2 [11] and CADD [12] in silico tools to predict pathogenicity, and Human Splicing Finder [13] to identify splicing effects. Variants were classified according to the ACMG guidelines [14], and confirmed by Sanger sequencing with segregation analysis when samples were available. Variants were submitted to the ClinVar database (SCV002761233, SCV002761243). Details of in silico predictions and ACMG/AMP classifications are described in Supplementary Table S1.

## Results

**Fig. 1:**
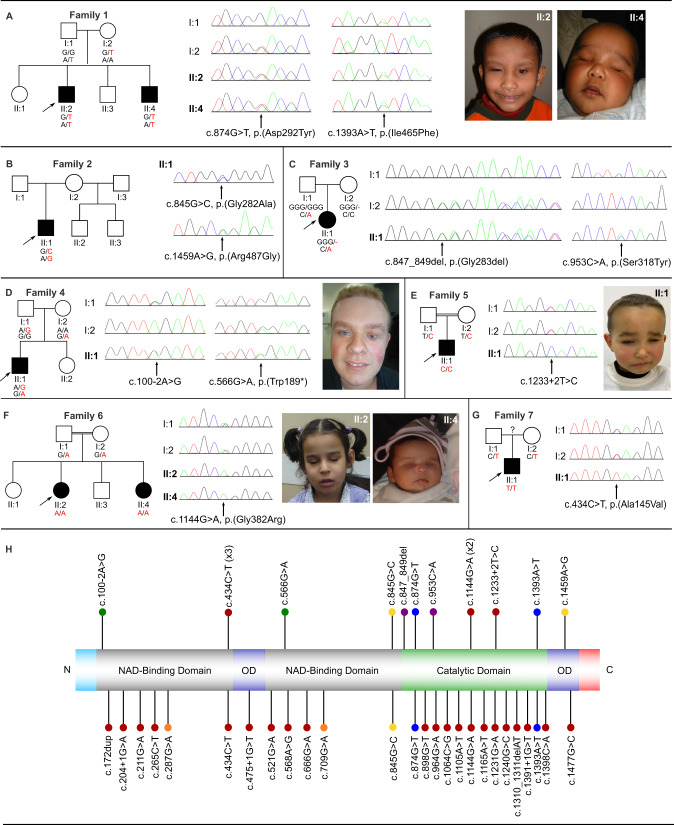
Clinical images, genetic findings and location of the *ALDH1A3* variants. **A**–**G** Pedigrees, genotypes and consented clinical images in families. **H** Schematic of ALDH1A3, its domains and variant locations reported to date. The variants identified in this study are listed above and previously reported variants below the domain structure. Compound heterozygous variant pairs are indicated in purple, orange, green, yellow and blue circles, homozygous variants in red circles. The previously reported variants also identified in our cohort are listed above and below the domain structure. The number of the reports are indicated in brackets.

We identified nine affected individuals with eleven biallelic *ALDH1A3* variants from seven unrelated families (Table 1 and Fig. 1). Unless stated, no other likely pathogenic variants relevant to the individuals’ phenotypes were identified.

**Table 1:** Summary of phenotypic and genetic findings.

Findings	Family 1, II:2	Family 1, II:4	Family 2, II:1	Family 3, II:1	Family 4, II:1	Family 5, II:1	Family 6, II:2	Family 6, II:4	Family 7, II:1
General Information									
Age	25 y	15 y	27 y	9 y	29 y	3 y	9 y	Birth	18 m
Gender	M	M	M	F	M	M	F	F	M
Ethnicity	Asian	Asian	White British	Mixed European Origin	Caucasian	Moroccan	Libyan	Libyan	Spanish
Consanguinity	No	No	No	No	No	Yes	Yes	Yes	NK
Genetic findings									
*ALDH1A3* variants^a^	c.874G>T, p.Asp292Tyr; c.1393A>T, p.Ile465Phe	c.874G>T, p.Asp292Tyr; c.1393A>T, p.Ile465Phe	c.845G>C, p.Gly282Ala; c.1459A>G, p.Arg487Gly	c.847_849del, p.Gly283del; c.953C>A, p.Ser318Tyr	c.566G>A, p.Trp189*; c.100-2A>G	c.1233 + 2T>C	c.1144G>A, p.Gly382Arg	c.1144G>A, p.Gly382Arg	c.434C>T, p.Ala145Val
Growth									
Birth weight (kg)	3.45	2.38	2.77	3.1	3.2	3.6	3.3	3.77	NK
Height (cm, centile)	165.5, 2nd–9th (24 y)	156, 9th (14 y)	NK	48.5; 25th (birth)	172; 25th (25 y)	51; 50th (birth)	123; 9th (9 y)	50; 50th (birth)	NK
Weight (kg, centile)	76.5, 75th–91st (24 y)	35.6, 2nd (14 y)	NK	NK	NK	NK	24; 9th–25th (9 y)	NK	NK
HC (cm, centile)	58, 91st–98th (24 y)	52.3, 0.4th–2nd (14 y)	NK	32.5; 9th (birth)	59.2; 91st–98th (25 y)	37; 75th (birth)	50.3; 10th (9 y)	35.5; 91st (birth)	NK
Ocular									
Anophthalmia	Bil	Bil	Bil	–	–	–	Bil	Bil	–
Microphthalmia	–	–	–	Bil	Bil	Bil (L > R)	–	–	Bil
Coloboma	–	–	–	Bil	–	R	–	–	Bil
Cataract	–	–	–	–	–	R	–	–	–
Other	Lower lid cyst (R)	–	Ocular cyst (Bil)	RD and nodule near retina with small calcifications	–	–	–	–	Abnormal ASM
Craniofacial									
Nasal anomalies	–	–	–	–	–	–	EN	–	WNB, EN, SP
SPF	–	–	–	–	–	–	+	+	+
DCM	–	–	–	–	–	–	–	+	+
Other	–	–	–	–	–	–	–	–	Telecanthus, Micrognathia, EF, EP, EUL, HF, FC
Developmental									
ID	–	+	+	–	–	–	+	–	–
Motor Delay	–	+	+	–	–	–	+	–	–
Speech Delay	–	Nonverbal	+	–	–	–	–	–	–
Autism	–	+	Autistic features	–	–	–	–	–	–
MRI findings	Normal	NK	Normal	NK	Normal	NK	Rudimentary ON and chiasm	Hypoplastic ON, Bil DL	NK
Other findings	Fistula-in-ano	–	Seizures	–	Crowded teeth	–	–	–	–

Variants are reported according to GRCh37/hg19.

*ASM* Anterior eye segment morphology, *Bil* Bilateral, *DCM* Downturned corners of mouth, *DL* Dysplastic lens, *EF* Epicanthic folds, *EN* Enlarged nares, *EP* Eyelid ptosis, *EUL* Everted upper lip, *F* Female, *FC* Full cheeks, *HC* Head circumference, *HF* High forehead, *ID* Intellectual disability, *L* Left, *M* Male, *m* months, *NK* Not known, *ON* Optic nerves, *R* Right, *RD* Retinal detachment, *SP* Short philtrum, *SPF* Short palpebral fissures, *WNB* Wide nasal bridge, *y* year.

^a^(NM_000693.4).

### Compound heterozygous variants

#### Family 1

The proband (II:2) had bilateral anophthalmia, a right lower lid cyst and normal development. His brother (II:4) presented with bilateral anophthalmia, and mildly delayed development until 2 years-of-age after which it gradually slowed. He was diagnosed with nonverbal autism at 3–4 years-of-age. Both brothers have a large sandal gap between the first and second toe. They carried compound heterozygous missense *ALDH1A3* variants (c.874G>T, p.(Asp292Tyr), maternal; c.1393A>T, p.(Ile465Phe), paternal). Both variants were absent in gnomAD and reported in DECIPHER. The proband had a normal SNP-Array and his brother had a normal array-CGH. A similar family has been published by Patel et al. [15], however it is unclear if this is the same family as described here.

#### Family 2

The proband (II:1) presented with bilateral anophthalmia with lower lid cysts. He had developmental delay and severe learning difficulties, delayed speech, autistic features, and tonic-clonic seizures (onset 13 years-of-age). He carries compound heterozygous missense variants (rs547918064, c.845G>C, p.(Gly282Ala), maternal, gnomAD MAF: 0.000019; c.1459A>G, p.(Arg487Gly), paternal, absent in gnomAD). The p.(Gly282Ala) variant was reported in the homozygous state by Alabdullatif et al. [16]. The parents are healthy with no family history of seizures.

#### Family 3

The proband (II:1) presented with bilateral microphthalmia and coloboma, bilateral retinal detachments with microcalcifications and vascularization at 1 month-of-age and normal development. She carries compound heterozygous variants: an inframe deletion of a highly conserved amino acid (c.847_849del, p.(Gly283del), maternal) and a missense (c.953C>A, p.(Ser318Tyr), paternal) variant. Both variants are absent in gnomAD. She had a normal array-CGH.

#### Family 4

The proband (II:1) presented with bilateral microphthalmia and normal development. We identified compound heterozygous nonsense (c.566G>A, p.(Trp189*), maternal, absent in gnomAD) and splice (rs1422193527, c.100-2A>G, paternal, gnomAD MAF: 0.00003183) variants.

### Homozygous variants

#### Family 5

The proband (II:1), born to consanguineous parents, presented with bilateral microphthalmia (extreme on the left) and additional coloboma and cataract of the right eye. He had normal development. He carries a homozygous splice variant (c.1233 + 2T>C, absent in gnomAD). His parents are heterozygous carriers. A paternal aunt had bilateral anophthalmia, but was unavailable for testing.

#### Family 6

The proband (II:2), born to consanguineous parents, presented with bilateral anophthalmia and mild intellectual delay. Her sister (II:4) had bilateral anophthalmia and normal development. Both carry a homozygous missense variant (c.1144G>A, p.(Gly382Arg), absent in gnomAD). Both parents were heterozygous carriers.

#### Family 7

The proband (II:1) presented with bilateral microphthalmia, iris and chorioretinal coloboma, abnormal anterior segment morphology and facial dysmorphic features, including high forehead, telecanthus, epicanthic folds, ptosis, full cheeks, everted upper lip, and micrognathia. He carries a homozygous missense variant (c.434C>T, p.(Ala145Val), gnomAD MAF: 0.000004). His parents are heterozygous carriers. There was no history of parental consanguinity, but the parents come from the same small town. Array-CGH was normal.

## Discussion

We report nine individuals from seven families with biallelic *ALDH1A3* variants (Fig. 1). In each case the variants were predicted disease-causing, with no other variants detected in genes associated with developmental eye disorders by WES/WGS/panel/CNV analysis. This study describes further cases with compound heterozygous *ALDH1A3* variants in A/M and highlights inter- and intrafamilial phenotypic variability.

Since many developmental eye genes are critical in the development of other organ systems, extraocular features are often observed in individuals with variants in developmental eye genes, such as *SOX2*, *OTX2* and *STRA6* [17]. Individuals with biallelic *ALDH1A3* variants have been previously reported with additional variable systemic features, including severe neurodevelopmental delay and autism, in addition to bilateral A/M [6, 9, 18]. Similarly, our nine cases consistently exhibited bilateral A/M; two with facial dysmorphic features (Families 6 and 7), and three also manifesting neurodevelopmental anomalies, including intellectual disability (Families 1, 2 and 6), autism (Families 1 and 2) and seizures (Family 2). Importantly, while additional ocular features are frequently reported in *ALDH1A3* cases, our study represents the first report of the presence of cataract (Family 5). The facial dysmorphic features described in individuals with *ALDH1A3* variants include bilateral small palpebral fissures or blepharophimosis [8, 9, 19, 20], which is often seen in individuals with small eye sockets secondary to severe A/M, irrespective of the genetic cause. However, broad eyebrows, synophrys and high arched palate are also reported in some cases [8]. Interestingly, one of our cases (Family 7, II:1) displayed multiple additional dysmorphic features, although it remains unclear if these are related to the *ALDH1A3* variants.

*ALDH1A3* is a member of the retinoic acid pathway, encoding aldehyde dehydrogenase involved in oxidation of retinaldehyde to retinoic acid (RA). RA levels are tightly regulated during embryonic development and are essential for normal eye morphogenesis [4]. Including this study, 32 pathogenic *ALDH1A3* variants have been reported: 18 missense, 7 splicing, 4 nonsense, 2 frameshift and 1 inframe deletion. Some of the missense (p.(Arg89Cys), p.(Ala493Pro), p.(Arg96His), p.(Gly237Arg)) and nonsense (p.(Lys190*), p.(Lys389*)) variants have been shown to impair protein production and cause loss of function and were reported in patients with variable additional neurodevelopmental phenotypes in the A/M spectrum [6, 19, 21].

To date, even with the growing evidence from cases, there is no consistent genotype-phenotype correlation. The majority of variants (16) are located in the catalytic domain, followed by the NAD binding domains (13) and the oligomerization domains (3) (Fig. 1). However, the location of variants does not appear to correlate with distinct phenotypic features or differences in severity. Furthermore, there is also striking inter- and intrafamilial variation even for the same variants. For example, Roos et al. [9] described neurodevelopmental intrafamilial variability in a large consanguineous family with microphthalmia/coloboma and a homozygous *ALDH1A3* variant (p.(Cys174Tyr)). Similarly, the affected brothers in Family 1, carrying the same compound heterozygous variants, had variable phenotypes: the older brother had isolated bilateral anophthalmia with normal intellect while the younger had severe neurodevelopmental delay. Furthermore, of the two affected sisters presenting with bilateral anophthalmia and facial dysmorphic features in Family 6 (p.(Gly382Arg)) the proband has mild intellectual delay whereas her younger sister has normal cognition. Interestingly, the same variant has been previously reported in four affected members of a family who presented with bilateral anophthalmia and facial dysmorphism, but normal psychomotor development [8], bringing into question the link between *ALDH1A3* variants and intellectual and developmental delay. In addition, the proband in Family 7 has the same variant (p.(Ala145Val)) that had been previously described in individuals from 3 independent families and a simplex case, all with isolated bilateral microphthalmia [5, 22], whereas he had some additional facial dysmorphic features. Therefore, the presence of inter- and intrafamilial variability suggests a more complex interplay between *ALDH1A3* variants and genetic and/or environmental factors, as might be expected for such a fundamentally important gene.

In conclusion, we present the clinical and genetic analysis of nine cases with biallelic *ALDH1A3* variants from seven families. Our data confirms that pathogenic *ALDH1A3* variants are consistently associated with bilateral A/M and highlights additional susceptibility to neurodevelopmental manifestations, with significant intra- and interfamilial variability. Moreover, one individual displayed microphthalmia with coloboma and cataract, broadening the ocular phenotype, and suggesting that it would be important to include this gene on cataract gene panels. Finally, the identification of four families with compound heterozygous variants underscores the importance of *ALDH1A3* screening in nonconsanguineous families.

### Supplementary information

Supplemental Table 1

## Data Availability

Data will be made available upon reasonable request. Variants were submitted to ClinVAR database (SCV002761233, SCV002761243).
