# Correction: Clinical and genetic analysis further delineates the phenotypic spectrum of *ALDH1A3*-related anophthalmia and microphthalmia

**DOI:** 10.1038/s41431-023-01363-3

**Published:** 2023-04-28

**Authors:** Yesim Kesim, Fabiola Ceroni, Alejandra Damián, Fiona Blanco-Kelly, Carmen Ayuso, Kathy Williamson, Véronique Paquis-Flucklinger, Dorine A Bax, Julie Plaisancié, Claudine Rieubland, Mostafa Chamlal, Marta Cortón, Nicolas Chassaing, Patrick Calvas, Nicola K Ragge

**Affiliations:** 1 Department of Biological and Medical Sciences, Faculty of Health and Life Sciences, Oxford Brookes University, Oxford, UK; 2 Department of Pharmacy and Biotechnology, University of Bologna, Bologna, Italy; 3 Department of Genetics & Genomics, Instituto de Investigación Sanitaria-Fundación Jiménez Díaz University Hospital, Universidad Autónoma de Madrid (IIS-FJD, UAM), Madrid, Spain; 4 Centre for Biomedical Network Research on Rare Diseases (CIBERER), Madrid, Spain; 5 MRC Human Genetics Unit, MRC Institute of Genetics and Molecular Medicine, University of Edinburgh, Western General Hospital, Edinburgh, UK; 6 Department of Medical Genetics, Nice Teaching Hospital, Nice, France; 7 INSERM U1214, ToNIC, Université Toulouse III, Toulouse, France; 8 Centre de Référence des Affections Rares en Génétique Ophtalmologique CARGO, Site Constitutif, CHU Toulouse, Toulouse, France; 9 Department of Human Genetics, Inselspital, Bern University Hospital, University of Bern, Bern, Switzerland; 10 Department of Pediatrics, RAZI-CLINIC Hospital, Tangier, Morocco; 11 Department of Clinical Genetics, West Midlands Regional Clinical Genetics Service and Birmingham Health Partners, Birmingham Women’s and Children’s Foundation Trust, Birmingham, UK

**Keywords:** Disease genetics, Neurodevelopmental disorders

Correction to: *European Journal of Human Genetics* 10.1038/s41431-023-01342-8, published online 31 March 2023

In this article, due to a typesetting mistake, the layout of Table 1 was wrong and it appeared incorrectly only in the article PDF; the table should have appeared as shown below.

**Table 1:** Summary of phenotypic and genetic findings.

Findings	Family 1, II:2	Family 1, II:4	Family 2, II:1	Family 3, II:1	Family 4, II:1	Family 5, II:1	Family 6, II:2	Family 6, II:4	Family 7, II:1
General Information									
Age	25 y	15 y	27 y	9 y	29 y	3 y	9 y	Birth	18 m
Gender	M	M	M	F	M	M	F	F	M
Ethnicity	Asian	Asian	White British	Mixed European Origin	Caucasian	Moroccan	Libyan	Libyan	Spanish
Consanguinity	No	No	No	No	No	Yes	Yes	Yes	NK
Genetic findings									
*ALDH1A3* variants^a^	c.874G>T, p.Asp292Tyr; c.1393A>T, p.Ile465Phe	c.874G>T, p.Asp292Tyr; c.1393A>T, p.Ile465Phe	c.845G>C, p.Gly282Ala; c.1459A>G, p.Arg487Gly	c.847_849del, p.Gly283del; c.953C>A, p.Ser318Tyr	c.566G>A, p.Trp189*; c.100-2A>G	c.1233 + 2T>C	c.1144G>A, p.Gly382Arg	c.1144G>A, p.Gly382Arg	c.434C>T, p.Ala145Val
Growth									
Birth weight (kg)	3.45	2.38	2.77	3.1	3.2	3.6	3.3	3.77	NK
Height (cm, centile)	165.5, 2nd–9th (24 y)	156, 9th (14 y)	NK	48.5; 25th (birth)	172; 25th (25 y)	51; 50th (birth)	123; 9th (9 y)	50; 50th (birth)	NK
Weight (kg, centile)	76.5, 75th–91st (24 y)	35.6, 2nd (14 y)	NK	NK	NK	NK	24; 9th–25th (9 y)	NK	NK
HC (cm, centile)	58, 91st–98th (24 y)	52.3, 0.4th–2nd (14 y)	NK	32.5; 9th (birth)	59.2; 91st–98th (25 y)	37; 75th (birth)	50.3; 10th (9 y)	35.5; 91st (birth)	NK
Ocular									
Anophthalmia	Bil	Bil	Bil	–	–	–	Bil	Bil	–
Microphthalmia	–	–	–	Bil	Bil	Bil (L > R)	–	–	Bil
Coloboma	–	–	–	Bil	–	R	–	–	Bil
Cataract	–	–	–	–	–	R	–	–	–
Other	Lower lid cyst (R)	–	Ocular cyst (Bil)	RD and nodule near retina with small calcifications	–	–	–	–	Abnormal ASM
Craniofacial									
Nasal anomalies	–	–	–	–	–	–	EN	–	WNB, EN, SP
SPF	–	–	–	–	–	–	+	+	+
DCM	–	–	–	–	–	–	–	+	+
Other	–	–	–	–	–	–	–	–	Telecanthus, Micrognathia, EF, EP, EUL, HF, FC
Developmental									
ID	–	+	+	–	–	–	+	–	–
Motor Delay	–	+	+	–	–	–	+	–	–
Speech Delay	–	Nonverbal	+	–	–	–	–	–	–
Autism	–	+	Autistic features	–	–	–	–	–	–
MRI findings	Normal	NK	Normal	NK	Normal	NK	Rudimentary ON and chiasm	Hypoplastic ON, Bil DL	NK
Other findings	Fistula-in-ano	–	Seizures	–	Crowded teeth	–	–	–	–

Variants are reported according to GRCh37/hg19.

*ASM* anterior eye segment morphology, *Bil* bilateral, *DCM* downturned corners of mouth, *DL* dysplastic lens, *EF* epicanthic folds, *EN* enlarged nares, *EP* eyelid ptosis, *EUL* everted upper lip, *F* female, *FC* full cheeks, *HC* head circumference, *HF* high forehead, *ID* intellectual disability, *L* left, *M* male, *m* months, *NK* not known, *ON* optic nerves, *R* right, *RD* retinal detachment, *SP* short philtrum, *SPF* short palpebral fissures, *WNB* wide nasal bridge, *y* year.

^a^(NM_000693.4).

The original article has been corrected.

