# Supplementary material for: Clinical and genetic analysis further delineates the phenotypic spectrum of ALDH1A3-related anophthalmia and microphthalmia
Source: Eur J Hum Genet. 2023 Mar 31;31(10):1175–80. doi: 10.1038/s41431-023-01342-8 (PMC10545824; doi:10.1038/s41431-023-01342-8)
Supplement: Supplementary file 1 — Supplemental Table 1 [file 41431_2023_1342_MOESM1_ESM.docx]

**Table S1**

| **Family ID** | **Zygosity** | **cDNA** | **Protein**  **(Domain)** | **Variant ID**  **(Frequency)** | **SIFT** | **Polyphen** | **CADD** | **Human Splicing Finder** | **ACMG**  **Criteria** | **Class** | **Previously reported variants** |
| --- | --- | --- | --- | --- | --- | --- | --- | --- | --- | --- | --- |
| Family 1 | Compound  heterozygous | c.874G>T | p.(Asp292Tyr)  (Catalytic) | NK | D | PD | 28 | - | PM2, PP1*, PP2, PP3 | VUS | Patel *et al.* 2019 |
|  |  | c.1393A>T | p.(Ile465Phe)  (Catalytic) | NK | D | PsD | 25.5 | Significant alteration of ESE/ESS motifs | PM2, PP1*, PP2, PP3 | VUS |  |
| Family 2 | Compound  heterozygous | c.845G>C | p.(Gly282Ala)  (NAD-Binding) | rs547918064  (0.00001989) | D | PD | 24 | - | PS1*, PM2, PP2, PP3 | LP | Alabdullatif *et al.* 2017 |
|  |  | c.1459A>G | p.(Arg487Gly)  (Oligomerization) | NK | D | PD | 24.9 | - | PM2, PM3*, PP2, PP3* | LP | - |
| Family 3 | Compound  heterozygous | c.847_849del | p.(Gly283del)  (Catalytic) | NK | NK | NK | NK | - | PM2*, PM4* | VUS | - |
|  |  | c.953C>A | p.(Ser318Tyr)  (Catalytic) | NK | D | PD | 24.3 | - | PM2, PP2, PP3* | VUS | - |
| Family 4 | Compound heterozygous | c.566G>A | p.Trp189*  (NAD-Binding) | NK | NK | NK | 41 | - | PVS1, PM2, PP3 | P | - |
|  |  | c.100-2A>G | -  (NAD-Binding) | rs1422193527  (0.00003183) | NK | NK | 34 | Alteration of the wt acceptor site of intron 1 | PVS1*, PM2*, PP3* | P | - |
| Family 5 | Homozygous | c.1233+2T>C | -  (Catalytic) | NK | NK | NK | 32 | Alteration of the wt donor site of intron 10 | PVS1*, PM2*, PP3* | P | - |
| Family 6 | Homozygous | c.1144G>A | p.(Gly382Arg)  (Catalytic) | rs1199864354  NK | D | PD | 30 | - | PS1*, PM2, PP1*, PP2, PP3 | LP | Abouzied *et al.* 2013 |
| Family 7 | Homozygous | c.434C>T | p.(Ala145Val)  (NAD-Binding) | rs754619607  (0.000004) | D | PD | 31 | - | PS1*, PM2, PP2, PP3 | LP | Aldahmesh *et al.* 2013 |

**Table S1: ACMG classifications and *in silico* predictions of the *ALDH1A3* variants identified in this study.** Variants are listed according to their cDNA and protein locations (NM_000693.4). For the frequency column, minor allele frequency has been retrieved from gnomAD database. All variants mentioned in this table are classified according to the ACMG guidelines using InterVAR (<https://wintervar.wglab.org/>). Any additional manually added ACMG evidence is marked with an asteriks. Abbreviations: D: Deleterious, ESE/ESS: Exonic Splicing Enhancer/Exonic Splicing Silencer, LP: Likely Pathogenic, NK: Not known, P: Pathogenic, PD: Probably Damaging, PsD: Possibly Damaging, VUS: Variant of uncertain significance, wt: wild type.
